# Right Porto-Ovarian H-Shunt for the Surgical Treatment of Symptomatic Portal Biliopathy: A Case Report and Literature Review

**DOI:** 10.1155/2009/152195

**Published:** 2009-06-25

**Authors:** Konstantinos Vasiliadis, Guido Engelmann, Peter Sauer, Jürgen Weitz, Jan Schmidt

**Affiliations:** ^1^Department of Surgery, University of Heidelberg, Im Neuenheimer Feld 110, 69120 Heidelberg, Germany; ^2^Department of Paediatrics, University of Heidelberg, Im Neuenheimer Feld 110, 69120 Heidelberg, Germany; ^3^Department of Gastroenterology, University of Heidelberg, Im Neuenheimer Feld 110, 69120 Heidelberg, Germany

## Abstract

Portal hypertension, especially when it is caused by extrahepatic portal vein thrombosis, is commonly followed by the development of an abnormal periportal and pericholedochal variceal network, which form a portal cavernoma. This may exert extrinsic pressure on the adjacent biliary ducts and gallblader, causing morphologic abnormalities, termed portal biliopathy, which is usually leading to asymptomatic cholestasis, while less frequently it can be associated with obstructive jaundice, gallstone formation, and cholangitis. Endoscopic stone extraction can effectively treat portal biliopathy when cholangitis is associated with common bile duct stones. Portosystemic shunts are indicated in cases of disease recurrence as they can achieve regression of portal cavernoma and usually relieve symptomatic portal biliopathy. This case describes an alternative partial portosystemic shunt that utilizes the right ovarian vein as an autologous conduit for the surgical treatment of symptomatic portal biliopathy.

## 1. Introduction

Extrahepatic portal vein thrombosis (EPVT) is a common cause of portal hypertension (PH) and is commonly followed by the formation of several periportal and pericholedochal collaterals [1, 2]. The development of this variceal network within the hepatic pedicle forms a portal cavernoma (PC) [3], which may exert extrinsic pressure on the adjacent biliary ducts, causing morphologic biliary abnormalities, termed portal biliopathy (PB) [4]. Usually, PB is leading to asymptomatic cholestasis, while less frequently is associated with obstructive jaundice, gallstone formation, and cholangitis [4, 5]. Endoscopic sphincterotomy and stone extraction can effectively treat PB when cholangitis is associated with common bile duct stones while total portosystemic shunt surgery is indicated when biliary obstruction is recurrent and/or progressive [6, 7]. Portal biliopathy poses a challenging therapeutic problem, as the presence of both PC and PH represent a significant obstacle to the surgical treatment of biliary obstruction. This case describes an alternative portosystemic shunt that utilizes the right ovarian vein as an autologous conduit for the surgical treatment of recurrent symptomatic portal biliopathy. To our knowledge, this is the first case reported in the medical literature of portal vein thrombosis associated with elevated factor VIII levels, which was complicated by symptomatic portal biliopathy and was treated with an alternative portosystemic shunt.

## 2. Case Report

**Figure 1:**
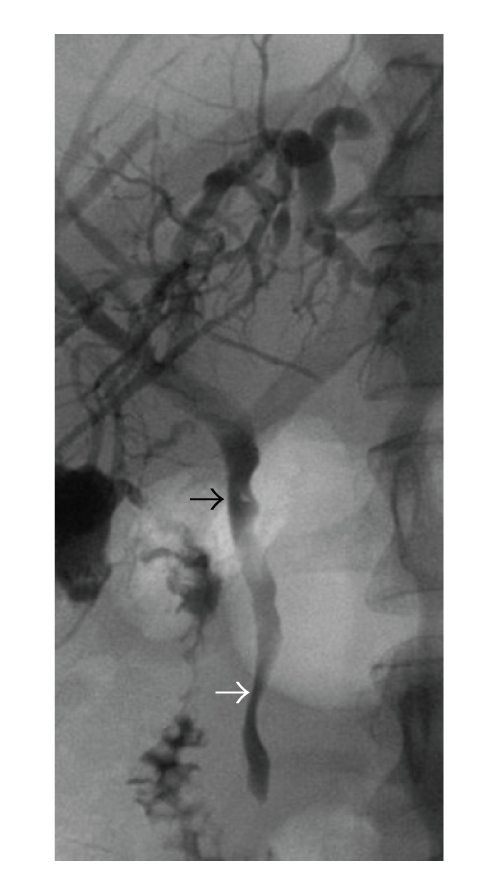
Tight and irregular stenosis of the common bile duct (white arrow) with upstream dilatation of its narrowed segment, in addition to multiple flat impressions. One solitary stone was also present (black arrow).

**Figure 2:**
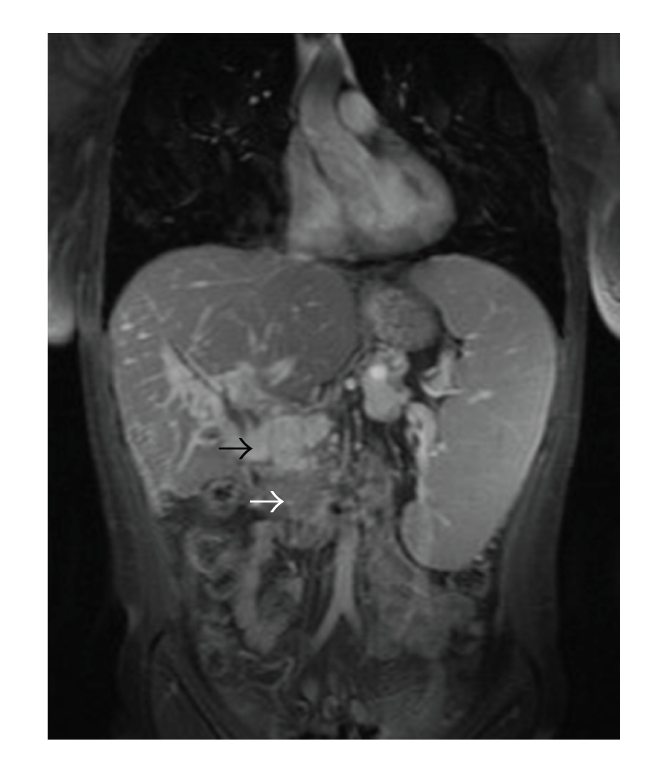
Coronal MRI T_2_ weighted image showing a solid tumor-like mass at the hepatic hilum (portal cavernoma), (white arrow) in addition to hyposignal (thrombus) at the level of mesentiricoportal confluence (black arrow).

**Figure 3:**
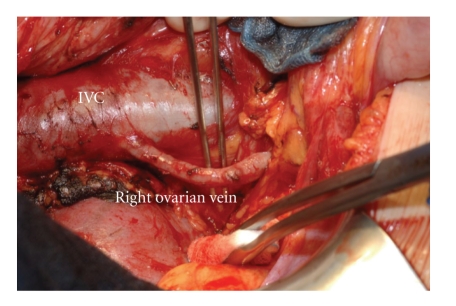
Operative photograph. The right ovarian vein, 6 mm in diameter draining into the IVC (inferior vena cava) just caudal to the right renal vein.

**Figure 4:**
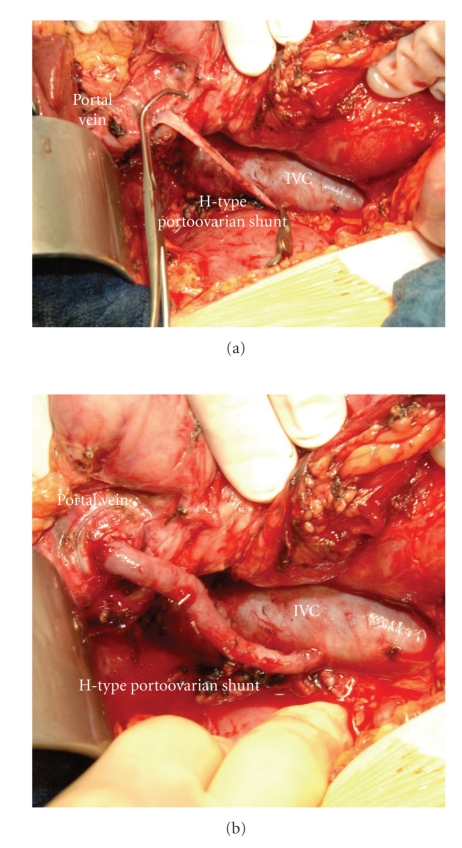
Operative photograph. Completion of the end-to-side right portoovarian anastomosis with a continuous 6-0 PDS suture, before (a) and after (b) clamp removal (PV: portal vein, IVC: inferior vena cava).

**Figure 5:**
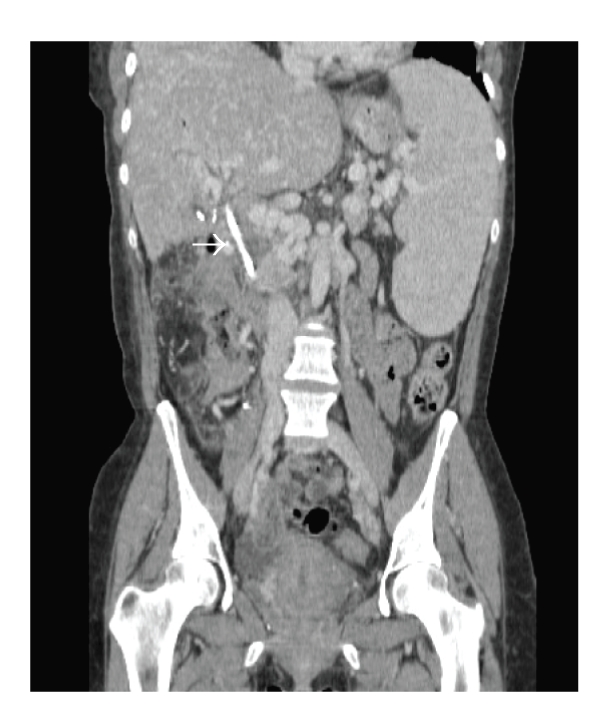
Postoperative coronal abdominal contrast enhanced CT scan shows opacification of the mesogonadal shunt (white arrow) confirming its patency.

A 39-year-old female patient was referred in April 2007 to the Surgical Department of the University Hospital of Heidelberg with a 3-year history of relapsing episodes of symptomatic portal biliopathy. The patient had a history of a hypercoagulable state owing to an abnormally elevated factor VIII level (factor VIII activity concentration 360% of normal, normal: 60–150), which was evident in October 2003 after an extensive diagnostic thrombophilic evaluation for idiopathic extrahepatic portal vein thrombosis complicated with PH. Extrahepatic PH was manifested with ascites, splenomegaly, and bleeding episodes from grade II oesophageal varices. The latter was treated by endoscopic sclerotherapy . At the time of initial presentation spiral computed tomography (CT) scan of the abdomen depicted a superior mesenteric and portal vein thrombosis in addition to cavernomatous transformation of the portal vein. Her family history was negative for thromboembolic events while she denied the use of oral contraceptive at the time of presentation. After diagnosis she was started on phenprocoumon for anticoagulation, which however discontinued because of episodes of variceal bleeding. A beta-blocker and nitrate were also administered for the treatment of portal hypertension. Six months following the initial presentation the patient started to complain of recurrent episodes of right upper quadrant pain, typically of biliary origin. Laboratory values showed a cholestatic pattern while abdominal ultrasound demonstrated a normal echogenicity of the liver as well as dilatation and irregularity of the common bile duct (CBD), which appeared surrounded by multiple tortuous collaterals in the region of the porta hepatis. The gallbladder appeared contracted and had multiple overlying collaterals, and contained stones and debris. Endoscopic retrograde cholangiopancreatography (ERCP) established the diagnosis of portal biliopathy by revealing irregular outline of the CBD in addition to several flat impressions, presumably caused by venous collaterals (Figure 1). These findings were consistent with grade I portal biliopathy [2]. After papillotomy, complete bile duct stone clearance was accomplished, followed by the insertion of an Amsterdam type biliary endoprosthesis. Laboratory values of cholestatic pattern improved in the immediate post-ERCP period. She was treated with ursodesoxicholic acid and was scheduled for a repeat ERCP session after a three-month interval. However, two months later a relapsing episode of obstructive jaundice and cholangitis ensued necessitating a repeat ERCP session and stent change. During the last 3-years, multiple ERCP sessions and stent changes were required, because of relapsing attacks of cholangitis, without, however, a definitive improvement. The patient was therefore referred for evaluation to our department and after a thorough consultation with the patient, the gastroenterologist and haematologist a decision was made for an open cholecystectomy and a portosystemic shunt. Preoperative magnetic resonance imaging (MRI) of the abdomen showed persistence of superior mesenteric and portal vein thrombosis, and cavernous transformation of the portal vein (Figure 2). Laparotomy was performed through an L-incision. After entering the peritoneal cavity large, dilated, tortous collaterals were found at the porta hepatis and around the CBD, crossing over the hepatoduodenal ligament and the gallbladder. Multiple inflammatory adhesions between the omentum and the contracted gallbladder were also present. Cholecystectomy was performed in a fundus-first approach. After careful dissection, and ligation of several tortous collaterals, the vessels and cystic duct in Calot's triangle were identified and ligated. Following cholecystectomy, it was decided to proceed with a portosystemic shunt. The distal portal vein (PV) was carefully isolated by minimal dissection avoiding the cavernoma in the liver hilum. The anterior portion of the adjacent inferior vena cava (IVC) was also dissected at the site chosen for the portocaval anastomosis. It was at this stage decided to perform a PV and IVC pressure measurement. The PV pressure was measured 30 mm Hg, while IVC pressure 9 mm Hg. However, the creation of a tension free side-to-side portocaval shunt proved technically not possible because of approximation difficulty, owing to the long vertical distance between the distal PV and IVC. In a search for potential H-shunt options, and considering the fact that patient's thrombophilia would made the use of a synthetic graft prone to thrombosis, a large 6 mm in diameter right ovarian vein draining into the IVC just caudal to the right renal vein appeared to be of adequate size and quality to be utilized as an autologous conduit, to perform a portosystemic shunt (Figure 3). Indeed, approximately 8 cm of its length was mobilized and the right ovarian vein was divided and ligated distally. After systemic anticoagulation with heparin, the proximal end of the ovarian vein was anastomosed end-to-side to the distal portal vein with a continuous 6-0 PDS suture (Figures 4(a)  and 4(b)). After clamp removal, Doppler confirmed excellent flow, while the pressure gradient between PV and IVC was once again measured confirming a reduction of at least 57% (the PV pressure was measured 20 mm Hg, while IVC pressure 8 mm Hg). The right ovarian vein had a smooth curve under the duodenum when the retractors were relaxed. This technique allowed bypassing the thrombosed portion of the portal vein by avoiding dissection of the cavernoma in the liver hilum and related risk of massive hemorrhage. The patient had an uncomplicated postoperative course and an abdominal CT scan (Figure 5) performed prior to discharge home confirmed patency of the shunt, despite difficulty visualizing the shunt by US examination. Low-molecular weight heparin was administered subcutaneously for three weeks, as she was scheduled to undergo a new ERCP session and stent removal by the end of this period. Following ERCP and stent removal, phenprocoumon was administered for anticoagulation. The patient did not developed encephalopathy while she has had no further episodes of bleeding, jaundice, abdominal pain, or recurrent fever, and no therapeutic intervention was required during a 19-month follow-up period (Table 1), although morphologic biliary abnormalities seen at US tended to persist.

## 3. Discussion

Underlying specific coagulation defects are increasingly recognized as precipitating factors of extrahepatic portal vein thrombosis (EPVT) [8]. In fact, in many patients with EPVT, multiple thrombophilic conditions are present and these may play an important role in the pathogenesis of EPVT [8]. However, elevated concentration of factor VIII has been rarely associated with this disorder [8–13]. 

Regardless of specific causative mechanisms, EPVT comes usually to clinical attention because of complications of PH [4, 6, 14]. Portal hypertension leads to the development of numerous venous collaterals that decompress into the systemic circulation. Bleeding from esophagogastric varices, which are commonly formed in the setting of EPVT, represent the most frequent manifestation of PH [15]. Furthermore, extensive collateral venous circulation can also develop from the epicholedochal venous plexus of Saint [16] and paracholedochal venous plexus of Petren [17] at the porta hepatis and bile ducts forming a portal cavernoma (PC) [4]. This may exert extrinsic pressure on biliary ducts, causing morphologic biliary abnormalities [5]. 

Portal biliopathy (PB), which is a recent terminology, describes these abnormal changes that include irregular strictures and dilatation of both extra and intrahepatic bile ducts, segmental dilated segments with a beaded appearance, ectasia and “pruning” of the intrahepatic bile ducts and varices of the gallbladder [18]. In this case patient's gallbladder had multiple overlying collaterals, while the CBD appeared dilated with an hourglass appearance upstream to PC. 

Khuroo et al. [19] have speculated that the abnormal CBD changes that develop in patients with PB could form because of vascular injuries at the time of portal vein thrombosis. They claimed that an extension of the thrombotic process might cause ischemic necrosis of bile ducts and subsequent stricture formation, caliber irregularities of biliary ducts and cholangiectases. On the other hand Webb and Sherlock [20] postulated that mechanical compression by engorged venous collaterals could be responsible for the development of symptomatic PB. Nevertheless, it should be noted that these and other different pathogenetic hypotheses are not mutually exclusive, and that each of various theories explain in part biliary and/or gallblader involvement in patients with PB [2]. In this case the presence of biliary abnormalities combined with biliary colic, in the absence of biliary symptoms before EPVT and cavernous transformation of the portal vein, is indicative of a potential role for biliary stasis. 

Portal biliopathy secondary to PC although quite common among patients with EPVT remains, however, a very rare and usually asymptomatic entity [18]. The majority of patients present asymptomatic cholestasis and demonstrate the characteristic changes of PB only on ERCP and/or MRCP [19, 21]. Several studies suggest that the prevalence of PB secondary to PC can range from 80% to 93% [5, 19, 21]. Rarely, biliary changes in patients with PB become significant to give rise to overt obstructive jaundice and contribute to the development of cholelithiasis and choledocholithiasis [18]. Upper quadrant pain typically of biliary origin, recurrent fever with chills and jaundice alone or in combination suggest the possibility of symptomatic PB. In this case the patient had neither biliary symptoms nor gallstone disease at onset however; during follow-up she developed recurrent attacks of cholecystitis and cholangitis. 

At present the management of PB is selective and is directed only to symptomatic patients [7, 18]. Asymptomatic patients do not require any treatment, especially if the liver function tests are normal. Choledocholithiasis, a known complication of PB in patients with EPVT, can be managed by endoscopic sphincterotomy (ES) and stone extraction [2]. Notwithstanding this, biliary changes often remaining unrelieved and chances of recurrent stones remain high. Thereby, the presence of strictures and/or significant abnormalities of CBD often necessitate the insertion of a biliary endoprosthesis [6]. This endoprosthesis often needs to be changed and sometimes, multiple ERCP sessions are required to keep the lumen patent, as happened in the present case. In regards to the effectiveness of ERCP and ES in the treatment of symptomatic PB it should be noted that a limited number of cases have documented successful treatment of symptomatic PB with only the insertion of a plastic stent [22].

In the presence of recurrent and/or progressive PB not amenable to endoscopic therapy, portosystemic shunt surgery decompressing portal circulation and causing regression of varices is indicated to relieve biliary stasis and prevent further stone formation [7]. However, operative management of these patients poses a challenging dilemma, as the presence of both PC and PH represent a significant obstacle to surgery. Indeed, dissection of the hepatic pedicle in the presence of PC can lead to life threatening hemorrhage [4, 7, 18]. Furthermore, the presentation of PB can be similar to cholangiocarcinoma [23] or sclerosing cholangitis [21] raising serious diagnostic issues that could lead to major errors in the management of this entity.

A variety of procedures have been used to treat symptomatic PB, however the optimal therapeutic strategy remains controversial. The first case of portosystemic shunt for symptomatic PB was reported by Choudhuri et al. [24]. They treated successfully symptomatic PB by performing a splenectomy and proximal splenorenal shunt. To date, 30 patients have been treated for symptomatic PB with a portosystemic anastomosis [4, 7, 24–28]. A mesocaval shunt with jugular vein interposition was the most frequent approach in pediatric patients while splenectomy and proximal splenorenal anastomosis was the most frequently performed operation in adults. In the most recently reported study, Vibert et al. [7] treated successfully 10 patients with symptomatic PB by performing a splenorenal anastomosis with internal jugular interposition graft via a retroperitoneal approach. In the above-mentioned study the authors claimed that the principal benefit of this approach was that the formation of peritoneal adhesions that could complicate a potentially necessary subsequent biliodigestive bypass is avoided.

In the present case the surgical strategy aimed primarily to decompress PC after performing an open cholecystectomy with the intent to relieve symptoms of biliary colic and prevent gallstone disease related complications. A mesocaval shunt was not an option because of superior mesenteric vein thrombosis, while a direct side-to-side portacaval shunt proved not possible because of complicated anatomy. Thereby, the right ovarian vein was successfully used as an in situ conduit to create an H-type portosystemic shunt. This alternative procedure created effectively a partial shunt offering additional shunt options in a patient with complicated anatomy and serious co-morbid conditions, while in parallel; it proved to be equivalent in function to the classic H-type portacaval shunt. Furthermore, this right portoovarian shunt was only partially diverting it using an autologous tissue, and was relatively easy to perform requiring only a single anastomosis with minimal dissection. In conclusion, this report describes the usefulness of an alternative type of portosystemic shunt that may be considered in patients with symptomatic portal biliopathy having the appropriate anatomy and limited shunt options because of serious comorbid conditions. 

## Figures and Tables

**Table 1:** Clinical course of the patient.

Date	Pathological condition	Management
10.2003	Idiopathic extrahepatic portal and superior mesenteric vein thrombosis, because of elevated factor VIII level, complicated with portal hypertension	Administration of phenprocoumon for anticoagulation (discontinued because of episodes of variceal bleeding), and beta-blocker and nitrate for portal hypertension
12.2003	Bleeding episodes from grade II oesophageal varices	Endoscopic sclerotherapy
04.2004	Grade I portal biliopathy	Endoscopic complete bile duct stone clearance and insertion of a biliary endoprosthesis, administration of ursodesoxicholic acid
06.2004–02.2007	Numerous relapsing episodes of symptomatic portal biliopathy	Multiple ERCP sessions and stent changes without a definitive improvement
04.2007	Symptomatic portal biliopathy- decision making for surgical management	Open cholecystectomy and an alternative portosystemic shunt (right porto-ovarian H-shunt)
05.2007–present	None. The patient did not developed encephalopathy while she has had no further episodes of bleeding, jaundice, abdominal pain, or recurrent fever	No therapeutic intervention was required
